# Machine learning with the hierarchy‐of‐hypotheses (HoH) approach discovers novel pattern in studies on biological invasions

**DOI:** 10.1002/jrsm.1363

**Published:** 2019-08-05

**Authors:** Masahiro Ryo, Jonathan M. Jeschke, Matthias C. Rillig, Tina Heger

**Affiliations:** ^1^ Institute of Biology Freie Universität Berlin Berlin Germany; ^2^ Berlin‐Brandenburg Institute of Advanced Biodiversity Research (BBIB) Berlin Germany; ^3^ Leibniz‐Institute of Freshwater Ecology and Inland Fisheries (IGB) Berlin Germany; ^4^ Biodiversity Research/Systematic Botany University of Potsdam Potsdam Germany; ^5^ Restoration Ecology Technical University of Munich Freising Germany

**Keywords:** artificial intelligence, hierarchy‐of‐hypotheses approach, machine learning, meta‐analysis, synthesis, systematic review

## Abstract

Research synthesis on simple yet general hypotheses and ideas is challenging in scientific disciplines studying highly context‐dependent systems such as medical, social, and biological sciences. This study shows that machine learning, equation‐free statistical modeling of artificial intelligence, is a promising synthesis tool for discovering novel patterns and the source of controversy in a general hypothesis. We apply a decision tree algorithm, assuming that evidence from various contexts can be adequately integrated in a hierarchically nested structure. As a case study, we analyzed 163 articles that studied a prominent hypothesis in invasion biology, the enemy release hypothesis. We explored if any of the nine attributes that classify each study can differentiate conclusions as classification problem. Results corroborated that machine learning can be useful for research synthesis, as the algorithm could detect patterns that had been already focused in previous narrative reviews. Compared with the previous synthesis study that assessed the same evidence collection based on experts' judgement, the algorithm has newly proposed that the studies focusing on Asian regions mostly supported the hypothesis, suggesting that more detailed investigations in these regions can enhance our understanding of the hypothesis. We suggest that machine learning algorithms can be a promising synthesis tool especially where studies (a) reformulate a general hypothesis from different perspectives, (b) use different methods or variables, or (c) report insufficient information for conducting meta‐analyses.

## 1 INTRODUCTION

Research synthesis is an essential scientific endeavor that integrates and assesses disparate data, concepts, and/or theories to yield novel insights or explanations^1^ by consolidating collected evidence.^2^, ^3^ It is expected to contribute to fostering evidence‐based research, policy, and practice.^4^, ^5^

Hypotheses and ideas that express a simple, yet general, statement are attractive in scientific disciplines studying highly context‐dependent systems (e.g., medical, social, political, educational, and biological sciences). In these disciplines, such general hypotheses must be tested repeatedly from various perspectives, under different contexts, and using different methods. Consequently, it is often the case that some studies support the hypothesis, while others reject it. An example is the enemy release hypothesis, one of the most prominent hypotheses to explain biological invasions.^6^, ^7^, ^8^, ^9^, ^10^, ^11^, ^12^ The hypothesis posits that the absence of enemies in the exotic range of non‐native species determines their invasion success.^13^, ^14^ Several synthesis studies suggested that the validity of this hypothesis is dependent on the context, determined by species' identity, ecosystem type, ecological level, and test method.^8^, ^14^, ^15^, ^16^, ^17^

Synthesis is methodologically quite challenging under such a strong context‐dependency. Meta‐analysis (quantitative synthesis) is sometimes not applicable, particularly when studies addressing the same question use different methods or measure different response variables.^18^ Note that we refer to meta‐analysis as a statistical method combining the magnitude of the effect sizes from different data sets (following^19^, ^20^), although broader definitions also exist.^21^ On the other hand, various qualitative synthesis approaches that can deal with context‐dependency have recently emerged (eg, thematic synthesis, textual narrative synthesis, and framework synthesis; reviewed in Dixon‐Woods et al and Barnett‐Page^4^, ^22^), but these can retain considerable subjectivity and bias, such as “cherry‐picking” of preferable evidence.^19^, ^20^, ^23^ While these limitations are recognized and cannot be denied, policy‐makers and practitioners need evidence synthesis by any means to comprehend and solve urgent real‐world problems that are highly complex and usually lack mechanistic explanations.^4^, ^22^

**Figure 1:**
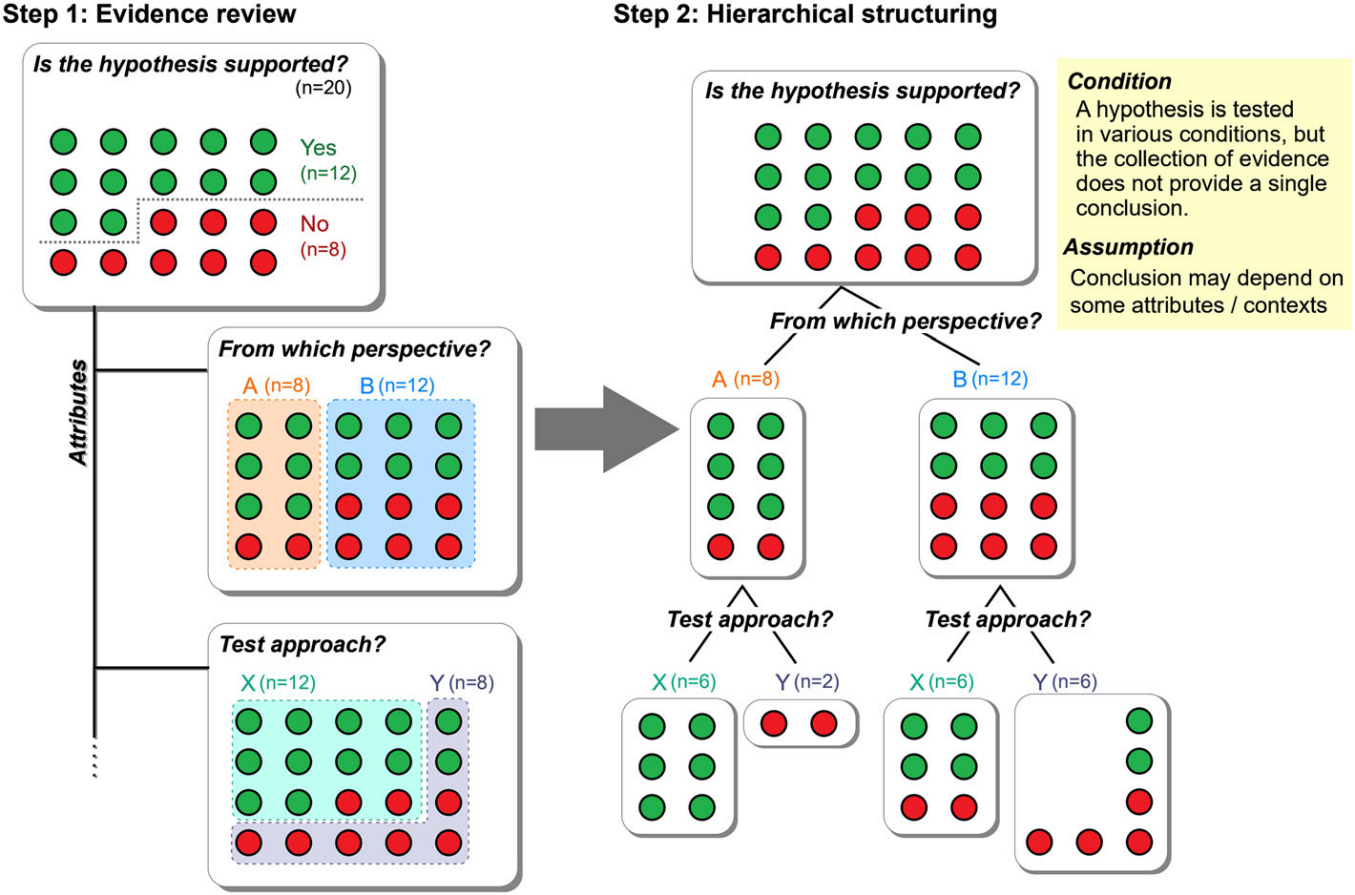
The hierarchy‐of‐hypotheses (HoH) approach as an evidence synthesis method. This approach is useful where a general hypothesis was tested repeatedly, and a single conclusion cannot be (or should not be) drawn from the collection of evidence because conclusions may be context‐dependent. First, some common attributes that differentiate the collection of evidence into some groups need to be found (step 1). Second, the collection is split into some groups in a hierarchically structured manner (step 2). Step 2 becomes difficult when many attributes are considered (ie, multidimensionality), and how the structure should look like may depend on researcher's perspective (cf. reproducibility) [Colour figure can be viewed at http://wileyonlinelibrary.com]

Recognizing the strong necessity to adequately deal with complexity and context‐dependency, we consider the *hierarchy‐of‐hypotheses* (HoH) approach as an attractive synthesis approach (Figure 1).^13^, ^14^, ^15^, ^24^ The HoH approach allows to integrate evidence addressing a given general hypothesis in various contexts by conceptually mapping the general hypothesis and the associated subhypotheses (ie, less general versions of the overarching hypothesis) and connecting them in a hierarchically nested fashion.^13^, ^14^, ^15^ The HoH approach is regarded as a hybrid of quantitative and qualitative synthesis, as it can combine the results of statistical tests using meta‐analyses or semi‐quantitative methods, while experts decide how to organize the hierarchical structure from the collected evidence. Thereby, for instance, one can readily detect specific contexts where the general hypothesis is more likely to be supported. Introducing the idea of hierarchical structuring enhances research synthesis, as it allows encompassing evidence from various contexts to gain an overview of the empirical base of the general hypothesis.

The HoH approach based on experts' judgement, however, is limited in reproducibility (ie, structure of the hierarchy can depend on expert perspective) and dimensionality (ie, only a few contexts can be considered at the same time because of limited human capacity). To overcome these limitations, we consider the use of machine learning, which is envisaged to play significant roles in research synthesis.^18^, ^25^ It is a set of statistical analysis tools in the field of artificial intelligence that find associations among variables automatically in a non‐parametric equation‐free manner. Machine learning with text mining can screen out articles relevant to the topic of interest from a large literature database automatically and instantaneously (eg, Cheng et al, Marshall et al, Zhaohan et al, and Przybyla et al^25^, ^26^, ^27^, ^28^). Moreover, the equation‐free modeling approach can discover unexpected patterns from collected data.^29^, ^30^, ^31^ For instance, in material science, machine learning discovered novel hypotheses on the crystallization of vanadium selenites from approximately 4000 single independent tests.^32^ Yet, the potential of machine learning in research synthesis remains largely elusive.

In this study, we show that machine learning is a promising tool in research synthesis for integrating collected evidence for discovering novel patterns and for finding the source of controversy in a general hypothesis. We apply a decision tree algorithm^33^, ^34^ for synthesizing evidence on the enemy release hypothesis as a case study. This method solves classification problems by splitting data into some groups hierarchically and hence applies the same principle as the HoH approach. The algorithm selects some explanatory variables that explain context‐dependency of the general hypothesis. We then evaluate if these variables were expected or surprising from an expert perspective by comparing the model structure with the original HoH built based on expert judgment.

## 2 MATERIALS AND METHODS

Two steps are taken for building an HoH. The first step is a literature review that includes the decisions on which studies to collect and how to categorize each study. The second step is building the hierarchy along with the decision on which of the information to use. For the second step, we will show two approaches: a first based on expert judgement and a second based on machine learning. Note that the first step and the expert judgement approach of the second step were conducted in the previous studies by some of the authors, Heger and Jeschke.^14^, ^15^ The data shown here are the ones assessed by them and presented in the book chapter.^15^

Step 1: Literature review and data collection

We summarize here how the database for analyzing evidence for the enemy release hypothesis was prepared (see Heger & Jeschke^14^, ^15^ for a more elaborate description). Heger and Jeschke^14^, ^15^ searched the Web of Science to identify studies addressing the enemy release hypothesis in its broad sense, ie, “the absence of enemies is a cause of invasion success.” Manual screening and assessing the eligibility resulted in 163 articles, which accounted for 248 subhypotheses (n = 248).

The authors categorized each subhypothesis based on the following aspects: hypothesis‐formulation (ie, how to make the overarching hypothesis less general and better testable by assigning a particular definition on each term in the general hypothesis); context‐dependence (ie, additional factors that may influence the conclusion); and test‐design (ie, detailed information on the designs of hypothesis testing).

Hypothesis‐formulation:

1. indicator to assess enemy release (damage the alien species received by enemies/infestation of the alien species/performance of the alien species);
2. type of comparison (alien and native species/alien species in their native range and in their invaded range/invasive and non‐invasive alien species/alien species' performances with and without enemies);
3. type of enemies (specialist/generalist);

Context‐dependence:

- geographic continent (eg, North America and Asia);
- studied habitat (terrestrial/freshwater/marine);

Test‐design:

- the number of focal species;
- the number of replicates;
- study method (observation [correlation]/experiment); and
- degree of realism (lab/enclosure/field).

These choices were made based on knowledge gained from a first screening of the articles concerning which research approaches have been used (1 to 3) and based on general knowledge about typical biases in ecology (4 to 9).

Then, the authors classified the conclusion of each subhypothesis based on the results and discussion into three categories—supported, questioned, or inconsistent. One may imagine the conclusion for each subhypothesis must be either supported or not (ie, questioned), based on statistical significance with frequentist statistics. The class “inconsistent” was assigned to a study that had tested a hypothesis using multiple approaches and obtained inconsistent indications: eg, through multiple experiments, statistical models, response variables, and geographic locations. For example, the class inconsistent is assigned to a study where a hypothesis was supported based on a species richness measure but questioned based on an abundance measure, or a hypothesis was supported for two out of four geographic regions. Note that in the previous studies,^14^, ^15^ the class inconsistent was termed as “undecided.”

Step 2a: Structuring the HoH with expert judgement (expert‐led HoH)

In Heger and Jeschke,^14^, ^15^ the authors structured an HoH using the collected evidence based on expert judgement (see there for a more elaborate description). Taking into account all categories at once was nearly impossible with human cognition, because how to structure an HoH becomes too flexible (eg, which variable should come first). Moreover, the collection of evidence would be split into too many subgroups if all categories were included. Therefore, the authors needed to decide which of the criteria should be used to build the hierarchy. After some trials, the authors agreed that up to three levels of subdivision can be understood readily and offer enough detailed contexts.

**Figure 2:**
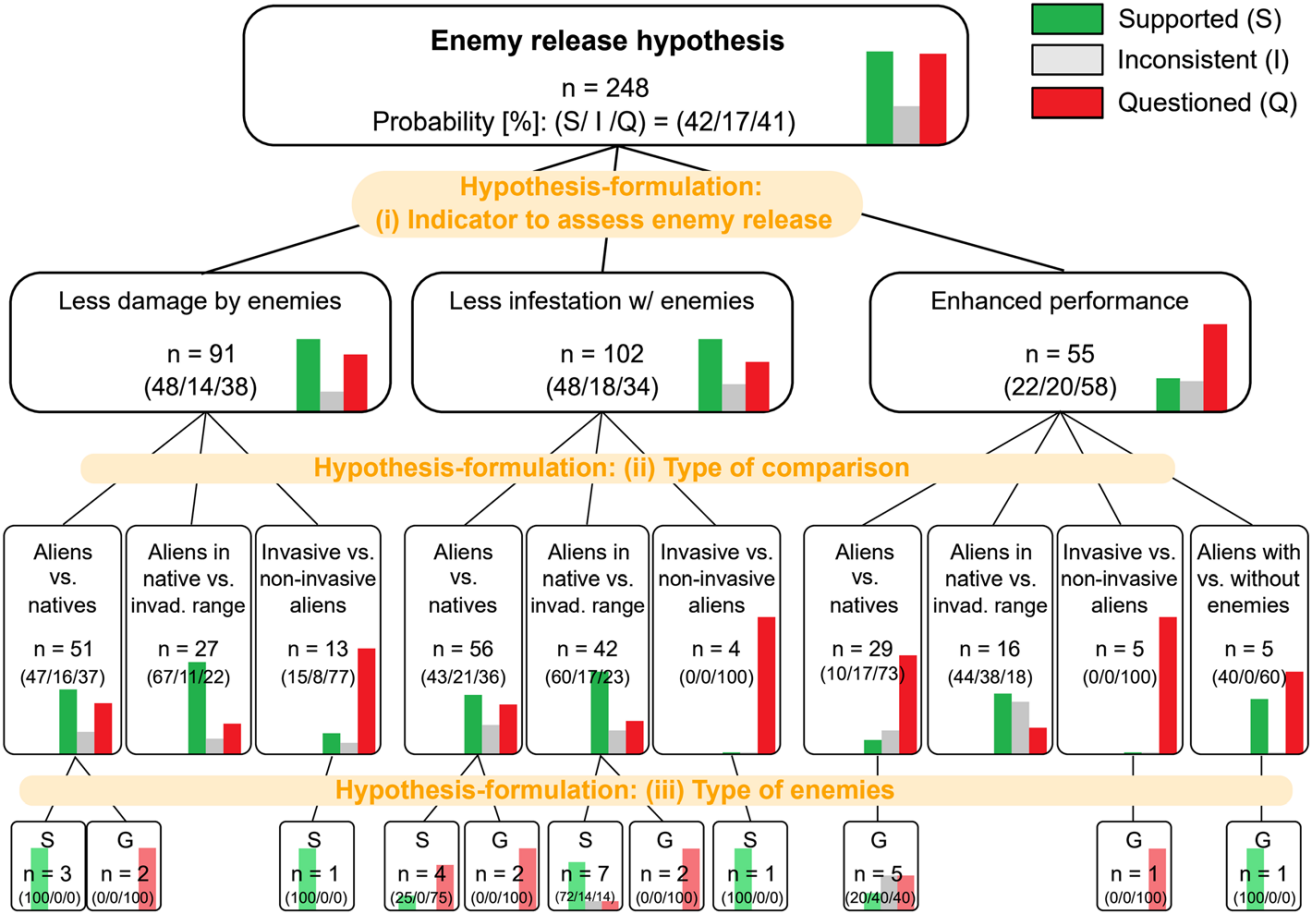
Hierarchy of hypotheses for enemy release hypothesis, built with the method described in Heger and Jeschke^14^ and updated with data from Heger & Jeschke.^15^ The hierarchical structure classifies the collected evidences based on three chosen criteria. The relative lengths of color bars and the numbers bracketed (supported/inconsistent/questioned, respectively) in each box indicate the relative proportion of conclusions [%] within the given context [Colour figure can be viewed at http://wileyonlinelibrary.com]

The authors also agreed that it is of major interest whether biological attributes may explain the context dependency. Therefore, the categories of hypothesis‐formulation, (1) to (3), were preferred over the others for building the hierarchical structure. Whether the level of support differs according to context and test design was less attractive to investigate. For each node, the numbers of studies that supported, questioned, or were inconsistent about the hypothesis were shown (see Figure 2). Note that decision criteria can depend on a researcher's perspective, as with any synthesis approaches.

Step 2b: Structuring the HoH with machine learning (machine‐led HoH)

For this study, we structured another HoH with the machine learning algorithm, conditional inference tree.^34^, ^35^ We used the entire set of Heger and Jeschke's predictors,^14^, ^15^ categories (1) to (9). The conditional inference tree is a decision tree algorithm suitable for detecting nonlinear, non‐additive patterns in a hierarchically structured manner.^33^ The algorithm partitions the samples into two subsamples by searching for a predictor variable and its threshold which differentiate the variability in the two subsamples the most. Once the sample is split, the algorithm attempts to further split each of the split subsamples. This trial is recursively done for each subsample until the algorithm cannot find a statistically significant split with any predictor and its threshold.^33^, ^34^, ^35^ When a predictor contains more than two categories, the split is done by finding the two groups of the categories that maximize among‐group variability.

The significance level was set to 0.05. In this study, the algorithm applied Kruskal‐Wallis tests for numeric predictors and Cochran‐Mantel‐Haenszel tests for categorical predictors.^35^ We used the “Partykit” package (version 1.1.1; Hothorn & Zeileis, 2016^36^) of *R* statistical computing (version 3.3.1; R Development Core Team, 2016^37^). The *R* script with the detailed descriptions and the data may be available at the github (https://github.com/masahiroryo/Machine-Learning-and-Hierarchy-of-Hypotheses).

## 3 RESULTS

**Figure 3:**
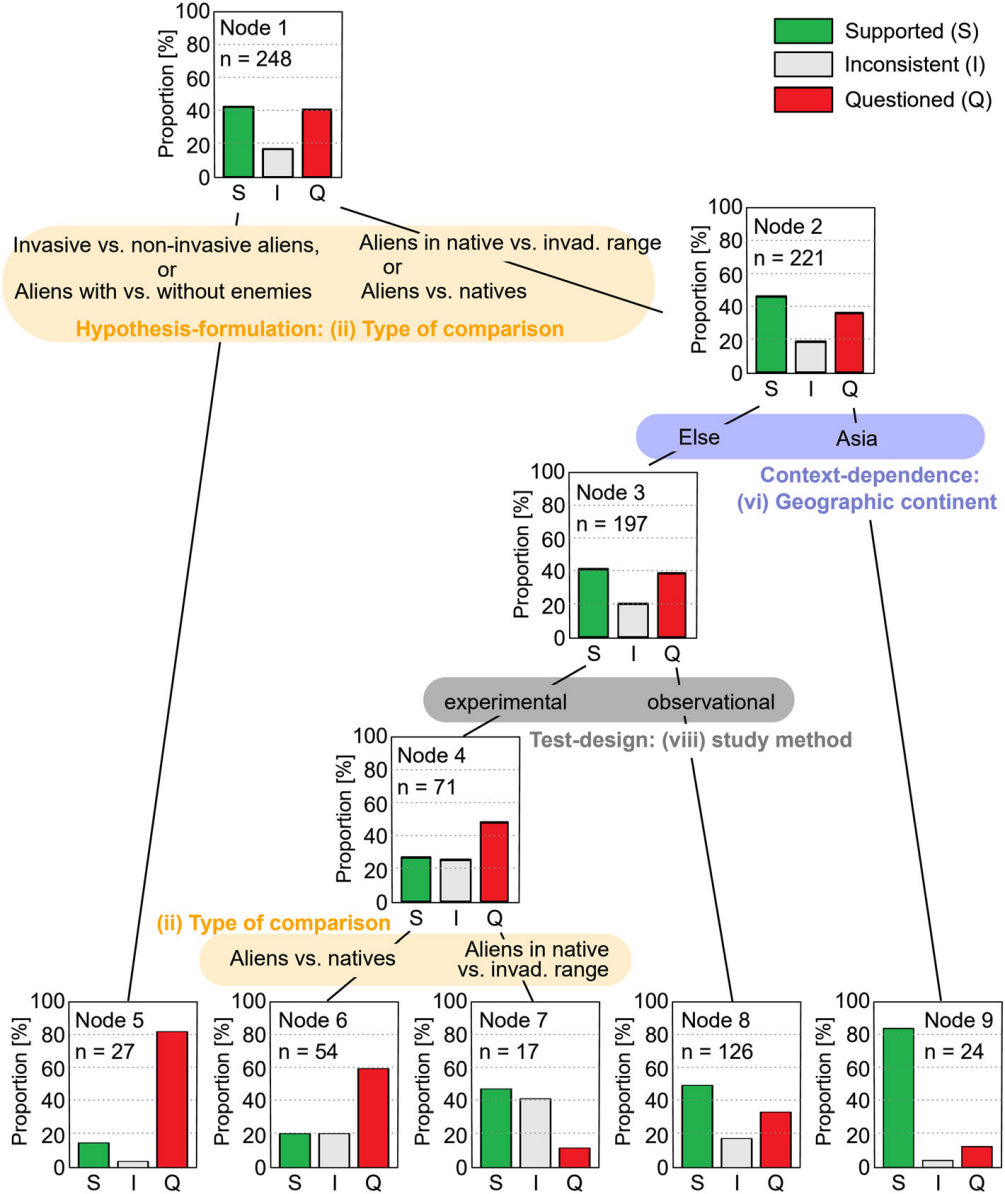
Hierarchy of hypotheses for enemy release hypothesis, built with the conditional inference tree machine learning algorithm. This analysis included the categories of hypothesis‐formulation, context‐dependency, and test‐design (nine predictors). Categories for split were automatically selected based on importance, and all splits are statistically significant (*α* = .05) [Colour figure can be viewed at http://wileyonlinelibrary.com]

Of 248 subhypotheses, there were about as many studies “supporting” as “questioning” the enemy release hypothesis (n = 105 and 101, respectively), with the remaining studies being inconsistent (n = 42). Both expert‐ and machine‐led HoHs showed different levels of support for each cluster of subhypotheses with a particular importance of hypothesis‐formulation, or more precisely, the type of comparison (2nd level split in Figure 2 and splits of nodes 1 and 4 in Figure 3). Among the types of comparison, only the studies that compared aliens in their native range and in invaded ranges tended to support the hypothesis (node 7 in Figure 3), but the others were more likely to question it (ie, invasive vs non‐invasive aliens [node 2], aliens with vs without enemies [node 2], and aliens vs natives [node 6]).

The two other criteria thought to be important in the expert‐led HoH (ie, indicator for enemy release and type of enemies) were not selected in the machine‐led HoH. Instead, geographic region (whether including Asia or not, node 2) and type of study design (either experiment or observation, node 3) appeared as significant factors. More than 80% of the studies related to Asia supported the hypothesis (node 9). Observational studies tended to support the hypothesis (node 8), whereas experimental studies tended to question it (node 4).

## 4 DISCUSSION

We demonstrated the ability of a machine learning algorithm with a hierarchical concept to perform synthesis of context‐rich evidence. In this approach, a first step is conducting a literature review with categorizing each study in a structured manner, and a second step is to apply a decision tree algorithm for its quantitative analysis. This synthesis approach can be useful where (a) studies reformulate a general hypothesis from different perspectives, (b) studies use diverse methods or variables,^18^ (c) many of them report insufficient information for conducting meta‐analysis.^38^, ^39^

A machine‐led HoH can be built instantaneously, in a reproducible manner, and it can detect unexpected patterns. Yet, this has some caveats: it sacrifices flexible imagination by experts in structuring an HoH. Given the strict quantitative rule, it may just find patterns that are out of scope. In addition, it does not consider the relative importance of each study as default (but weighting would be possible). A single study that conducted a test very rigorously can be clustered with less rigorous ones. Therefore, the expert‐led HoH approach (or another qualitative synthesis tool) should be applied to complement the machine‐led HoH approach. We believe that experts' intuition and creativity are important for directing the structure of the theoretical body, and for deciding what should be further investigated.

The most unexpected discovery was that more than 80% of the studies focused on Asia supported the enemy release hypothesis (node 9 in Figure 3). A study focus on Asia means that these empirical tests examined either (a) species native to Asian regions introduced to non‐Asian regions or (b) species non‐native to Asian regions introduced to Asian regions. We investigated whether either of the two features can explain the reason for the high support, but both features were evenly studied (13 case studies for each) and equally supportive. Therefore, the direction of invasion pathway,^40^ either species arriving from or to Asian regions, could not explain this pattern. We note that this finding does not necessarily imply any causal relationships and we cannot discard the possibility of a Type II error on this finding (ie, there is no true relationship, but it suggests statistical significance solely by chance). However, Asian regions have been largely understudied in invasion biology,^41^ and therefore, “Why Asia?” remains an important open question in invasion biology. Such novel pattern discovery using machine learning for generating a new question is useful in fields where theory and mechanistic understanding are still immature and context‐rich systems are studied.^29^, ^30^, ^31^, ^42^, ^43^

The structure of the machine‐led HoH differed from that of the expert‐led HoH, but this does not mean that machine learning algorithms suggest results totally unexpected by experts. Indeed, the results of the machine‐led HoH are in harmony with previous narrative syntheses about the enemy release hypothesis: (a) whether an alien species is invasive or not does not generally depend on whether it is released from its enemies or not (node 5 in Figure 3)^16^; (b) experimental evidence is more equivocal than observational (correlational) evidence (nodes 4 and 8)^8^; and (c) community studies (node 6) are less supportive than biogeographical studies (nodes 7 and 9).^16^ Our analysis supports these findings quantitatively and statistically, and these agreements corroborate the usefulness of machine learning for supporting expert judgement in research synthesis.

The particular benefits of employing machine learning over relying solely on expert judgment are the instantaneous speed for building an HoH, reproducibility ensured by the quantitative decision rules, and the ability of analyzing far more factors than a human expert. On the other hand, the expert‐led HoH is necessary if the literature analysis has the aim to answer specific questions. For example, the expert‐led HoH in our case revealed that species in many cases seem to be released from their enemies (less damage and less infestation), but that this release is not necessarily connected to a better performance of the species (low support for the respective hypothesis, see Figure 2). Moreover, the expert‐led HoH helps to discover gaps of knowledge. The expert‐led HoH (Figure 2), for example, can emphasize the necessity of more studies involving (a) experimental tests comparing aliens' statuses with vs without enemies and (b) specialist enemies. The first one is necessary to strictly test the enemy release hypothesis positing the absence of enemies as a cause of invasion success. The second one is also important, because given basic ecological knowledge, specialist enemies should play a key role in enemy release.^8^ Thus, both machine‐ and expert‐led HoHs can make valuable contributions to research synthesis: the former by identifying groups of cases in which a major hypothesis tends to be supported, and the latter by answering questions derived based on theoretical considerations and by identifying gaps of knowledge.

## CONFLICT OF INTEREST

The author reported no conflict of interest.

## Data Availability

The *R* script with detailed descriptions and the data is available at github (https://github.com/masahiroryo/Machine-Learning-and-Hierarchy-of-Hypotheses).

## References

[1] Pickett S , Kolasa J , Jones C . Ecological Understanding: The Nature of Theory and the Theory of Nature. 2nd ed. San Diego: Academic Press; 2007.

[2] Carpenter SR , Armbrust EV , Arzberger PW , et al. Accelerate synthesis in ecology and environmental sciences. Bioscience. 2009;59(8):699‐701. 10.1525/bio.2009.59.8.11

[3] Hackett EJ , Parker JN , Conz D , Rhoten D , Parker A . Ecology Transformed: The National Center for Ecological Analysis and Synthesis and the Changing Patterns of Ecological Research. Boston, MA: MIT Press; 2008.

[4] Dixon‐Woods M , Agarwal S , Jones D , Young B , Sutton A . Synthesising qualitative and quantative evidence: a review of possible methods. J Health Serv Res Policy. 2005;10(1):45‐53. 10.1258/1355819052801804 15667704

[5] Wyborn C , Louder E , Harrison J , et al. Understanding the impacts of research synthesis. Environ Sci Policy. 2018;86(October 2017):72‐84. 10.1016/j.envsci.2018.04.013

[6] Enders M , Hutt M‐T , Jeschke JM . Drawing a map of invasion biology based on a network of hypotheses. Ecosphere. 2018;9(3):e02146 10.1002/ecs2.2146

[7] Maron JL , Vilà M . When do herbivores affect plant invasion? Evidence for the natural enemies and biotic resistance hypotheses. Oikos. 2001;95(3):361‐373. 10.1034/j.1600-0706.2001.950301.x

[8] Keane RM , Crawley MJ . Exotic plant invasions and the enemy release hypothesis. Trends Ecol Evol. 2002;17(4):164‐170. 10.1016/S0169-5347(02)02499-0

[9] Mitchell CE , Power AOG . Release of invasive plants from fungal and viral pathogens. Nature. 2003;421(6923):625‐627. 10.1038/nature01317 12571594

[10] Torchin ME , Lafferty KD , Dobson AP , McKenzie VJ , Kuris AM . Introduced species and their missing parasites. Nature. 2003;421(6923):628‐630. 10.1038/nature01346 12571595

[11] Blumenthal D , Mitchell CE , Pysek P , Jarosik V . Synergy between pathogen release and resource availability in plant invasion. Proc Natl Acad Sci. 2009;106(19):7899‐7904. 10.1073/pnas.0812607106 19416888 PMC2674393

[12] Lowry E , Rollinson EJ , Laybourn AJ , et al. Biological invasions: a field synopsis, systematic review, and database of the literature. Ecol Evol. 2013;3(1):182‐196. 10.1002/ece3.431 PMC3568853 23404636

[13] Jeschke J , Gómez Aparicio L , Haider S , et al. Support for major hypotheses in invasion biology is uneven and declining. NeoBiota. 2012;14:1‐20. 10.3897/neobiota.14.3435

[14] Heger T , Jeschke JM . The enemy release hypothesis as a hierarchy of hypotheses. Oikos. 2014;123(6):741‐750. 10.1111/j.1600-0706.2013.01263.x

[15] Heger T , Jeschke JM . Enemy release hypothesis In: Jeschke JM, Heger T, eds. Invasion Biology. Hypotheses and Evidence. 1st ed. Boston, MA: CABI; 2018:92‐102.

[16] Colautti RI , Ricciardi A , Grigorovich IA , MacIsaac HJ . Is invasion success explained by the enemy release hypothesis? Ecol Lett. 2004;7(8):721‐733. 10.1111/j.1461-0248.2004.00616.x

[17] Liu H , Stiling P . Testing the enemy release hypothesis: a review and meta‐analysis. Biol Invasions. 2006;8(7):1535‐1545. 10.1007/s10530-005-5845-y

[18] Sutherland WJ , Wordley CFR . A fresh approach to evidence synthesis comment. Nature. 2018;558(7710):364‐366. 10.1038/d41586-018-05472-8 29925977

[19] Koricheva J , Gurevitch J , Mengersen K . Handbook of Meta‐Analysis in Ecology and Evolution. Princeton: Princeton University Press; 2013.

[20] Gurevitch J , Koricheva J , Nakagawa S , Stewart G . Meta‐analysis and the science of research synthesis. Nature. 2018;555(7695):175‐182. 10.1038/nature25753 29517004

[21] Glass GV . Primary, secondary, and meta‐analysis of research. Educ Res. 1976;5(10):3‐8. 10.1002/hlca.200390194

[22] Barnett‐page E , Thomas J . Methods for the synthesis of qualitative research: a critical review. BMC Med Res Methodol. 2009;9(59):1‐11. 10.1186/1471-2288-9-59 19671152 PMC3224695

[23] Cooper H , Hedges LV , Valentine JC . The Handbook of Research Synthesis and Meta‐Analysis. 2nd ed. New York: Russell Sage Foundation; 2009.

[24] Lokatis S , Jeschke JM . The island rule: an assessment of biases and research trends. J Biogeogr. 2018;45(2):289‐303. 10.1111/jbi.13160

[25] Cheng SH , Augustin C , Bethel A , et al. Using machine learning to advance synthesis and use of conservation and environmental evidence. Conserv Biol. 2018;32(4):762‐764. 10.1111/cobi.13117 29644722

[26] Marshall IJ , Noel‐Storr A , Kuiper J , Thomas J , Wallace BC . Machine learning for identifying randomized controlled trials: an evaluation and practitioner's guide. Res Synth Methods. 2018;Early View;9(4):602‐614. 10.1002/jrsm.1287 29314757 PMC6030513

[27] Zhaohan X , Tong L , Gary T , et al. A machine learning aided systematic review and meta‐analysis of the relative risk of atrial fibrillation in patients with diabetes mellitus. Front Physiol. 2018;9:835. 30018571 10.3389/fphys.2018.00835 PMC6037848

[28] Przybyła P , Brockmeier AJ , Kontonatsios G , et al. Prioritising references for systematic reviews with RobotAnalyst: a user study. Res Synth Methods. 2018;9(3):470‐488. 10.1002/jrsm.1311 29956486 PMC6175382

[29] Hochachka WM , Caruana R , Fink D , et al. Data‐mining discovery of pattern and process in ecological systems. J Wildl Manage. 2007;71(7):2427‐2437. 10.2193/2006-503

[30] Ryo M , Rillig MC . Statistically reinforced machine learning for nonlinear patterns and variable interactions. Ecosphere. 2017;8(11):e01976 10.1002/ecs2.1976

[31] Ryo M , Harvey E , Robinson CT , Altermatt F . Nonlinear higher order abiotic interactions explain riverine biodiversity. J Biogeogr. 2018;45(3):628‐639. 10.1111/jbi.13164

[32] Raccuglia P , Elbert KC , Adler PDF , et al. Machine‐learning‐assisted materials discovery using failed experiments. Nature. 2016;533(7601):73‐76. 10.1038/nature17439 27147027

[33] Breiman L , Friedman J , Stone CJ , Olshen RA . Classification and Regression Trees. Wadsworth, New York: Chapman and Hall/CRC; 1984.

[34] Hothorn T , Hornik K , Zeileis A . Unbiased recursive partitioning: a conditional inference framework. J Comput Graph Stat. 2006;15(3):651‐674.

[35] Hothorn T , Hornik K , van de Wiel MA , Zeileis A . A lego system for conditional inference. Am Stat. 2006;60(3):257‐263. 10.1198/000313006X118430

[36] Hothorn T , Zeileis A. A toolkit for recursive partitioning. 2016 http://partykit.r-forge.r-project.org/partykit. 10.1515/ijb-2015-0032 27227717

[37] R Development Core Team . R: A language and environment for statistical computing. 2016.

[38] Fidler F , En Chee Y , Wintle BC , Burgman MA , McCarthy MA , Gordon A . Metaresearch for evaluating reproducibility in ecology and evolution. Bioscience. 2017;67(3):282‐289. 10.1093/biosci/biw159 28596617 PMC5384162

[39] Parker TH , Forstmeier W , Koricheva J , et al. Transparency in ecology and evolution: real problems, real solutions. Trends Ecol Evol. 2016;31(9):711‐719. 10.1016/j.tree.2016.07.002 27461041

[40] Wilson JRU , Dormontt EE , Prentis PJ , Lowe AJ , Richardson DM . Something in the way you move: dispersal pathways affect invasion success. Trends Ecol Evol. 2009;24(3):136‐144. 10.1016/j.tree.2008.10.007 19178981

[41] Pyšek P , Richardson DM , Pergl J , Jarošík V , Sixtová Z , Weber E . Geographical and taxonomic biases in invasion ecology. Trends Ecol Evol. 2008;23(5):237‐244. 10.1016/j.tree.2008.02.002 18367291

[42] Bergmann J , Ryo M , Prati D , Hempel S , Rillig MC . Roots traits are more than analogues of leaf traits: the case for diaspore mass. New Phytol. 2017;216(4):1130‐1139. 10.1111/nph.14748 28895147

[43] Kelling S , Hochachka WM , Fink D , et al. Data‐intensive science: a new paradigm for biodiversity studies. Bioscience. 2009;59(7):613‐620. 10.1525/bio.2009.59.7.12

